# Expanding access to mental health care: a missing ingredient

**DOI:** 10.1016/S2214-109X(14)70029-4

**Published:** 2014-03-27

**Authors:** Helen Jack, Alan Stein, Charles R Newton, Karen J Hofman

**Affiliations:** University of Oxford, Department of Psychiatry, Oxford OX3 7JX, UK; University of Oxford, Department of Psychiatry, Oxford OX3 7JX, UK; MRC/Wits Rural Public Health and Health Transitions Research Unit [Agincourt], School of Public Health, Faculty of Health Sciences, University of the Witwatersrand, Johannesburg 2193, South Africa; MRC/Wits Rural Public Health and Health Transitions Research Unit [Agincourt], School of Public Health, Faculty of Health Sciences, University of the Witwatersrand, Johannesburg 2193, South Africa; PRICELESS SA [Priority Cost Effective Lessons in System Strengthening South Africa], School of Public Health, Faculty of Health Sciences, University of the Witwatersrand, Johannesburg 2193, South Africa

A tactical change in research and advocacy is needed urgently before mental health services will be made a priority in low-income and middle-income countries. Mental disorders and substance misuse are the greatest contributors to the global burden of disability, with 175·3 million years lost to disability annually—just under a quarter (22·9%) of the disability burden.^1^ Despite the great effect mental health disorders have on lives and livelihoods, and despite calls for action from researchers and clinicians, little has been done on a large scale to address mental health in low-income and middle-income countries. Budgets for mental health care are still around 0·5% of total health budgets in low-income countries, compared with more than 5% in high-income countries.^2^

Many of the specific interventions that researchers and clinicians laud publicly have not been implemented widely. For instance, leaders in global mental health have been calling for the integration of mental health into primary and chronic disease care for more than 40 years. Appeals for integration span from 1974, with a call by WHO’s Expert Committee on the Organization of Mental Health Services in Developing Countries,^3^ to 2013, in a series published in *PLoS Medicine*,^4^ to the present day, with WHO’s *Mental Health Action Plan 2013–2020*.^5^ Much has been said, but few integrated interventions have been rolled out at the national level or even piloted in low-income and middle-income countries.^6^

Global inaction could be attributable partly to failure to prove the economic costs of mental disorders in low-income and middle-income countries—not merely the cost of interventions but, importantly, the price and results of inaction. If funders and policymakers understood the full economic effect of mental disorders they might take a different view on investment in mental health care. We believe that the research community should focus attention on the costs of mental disorders and use the economic data for advocacy. This effort should encompass disorders in childhood and adolescence, because the costs of these disorders can persist into adulthood or be precursors of other disorders, making early intervention essential and cost-saving in the long term.^7^

The costs of failing to address mental health are high and mounting. Mental disorders cost low-income and middle-income countries US$870 billion every year,^8^ a cost that is estimated will more than double to US$2·1 trillion by 2030. These costs have substantial direct (supply-side expenses associated with treatment of mental disorders) and indirect (money lost or foregone because people with mental disorders and their caregivers are unable to work, receive education, or care for families) components. For instance, depression decreases adherence to antiretroviral treatment among people living with HIV,^9^ and, presumably, poses challenges for treatment of other chronic disorders, such as epilepsy, diabetes, and hypertension. Furthermore, mental disorders complicate interventions that entail regular appointments or lifestyle changes, including prevention of mother-to-child transmission—eg, HIV-positive women with post-partum depression are less likely to adhere to the strict course of HIV prophylaxis needed to keep their baby free from HIV.^10^ Compounding these difficulties is growing evidence showing that many chronic diseases—including HIV, diabetes, and epilepsy—are comorbid with mental disorders in low-income and middle-income countries.^11–13^ Resources spent on mental health could increase the effectiveness of interventions for chronic diseases.

Similarly, mental disorders harm other areas of society. Depression in adults raises the risk that their children will face various adverse effects.^14^ Parents suffering from depression, struggling even to leave the house, might be unable to work and need family members to stay home and care for them, trapping the family in a cycle of poverty that can exacerbate psychological distress.^15^ In a household survey from Ghana,^16^ severe psychological distress was associated with a 7% reduction in gross domestic product because of reduced work time, a loss greater than that attributable to malaria in a country that is malaria-endemic. People with untreated mental disorders can overwhelm public services such as welfare, law enforcement, and prisons; government spending in these areas could be curbed with better services for mental health. Data on the societal costs of entirely preventable exposures, including fetal alcohol syndrome and lead poisoning from paint or petroleum products, could ensure that efforts to prevent or manage intellectual disabilities in low-income and middle-income countries become a priority. The specific costs of mental disorders on the health, education, and economic systems of low-income and middle-income countries have not been thoroughly recorded, and more information is needed before we can fully understand and address this burden.

Data on the costs of mental disorders could motivate changes in mental health services. In the UK, economists and clinical researchers showed that increasing access to evidence-based psychological services (eg, cognitive behavioural therapy) would be nearly cost-neutral for the UK Government because of increased productivity and tax revenue and reduced social service costs (mainly health care and welfare benefits).^17^ The findings were used in a large public advocacy campaign that resulted in the UK Government committing publicly to investment in psychosocial treatments and, subsequently, nationwide scale-up of cognitive behavioural therapy.^17^ Similar, detailed country-specific economic evidence could affect policy in low-income and middle-income countries.

The ultimate concern is the human cost of mental health disorders—ie, the effects on the wellbeing of individuals, their families, and communities. If we care deeply about the quality of human life then we must think strategically and pragmatically about the tactics we use to bring about change in the mental health system. Economic data are simply one approach among many that researchers, practitioners, and advocates can use to help change how mental health is addressed in low-income and middle-income countries. We can no longer afford to ignore the costs because they are important for convincing governments and policymakers to act.
